# Supplementary figures and images for: Genome-wide survey of the F-box/Kelch (FBK) members and molecular identification of a novel FBK gene TaAFR in wheat
Source: PLoS One. 2021 Jul 22;16(7):e0250479. doi: 10.1371/journal.pone.0250479 (PMC8298115; doi:10.1371/journal.pone.0250479)

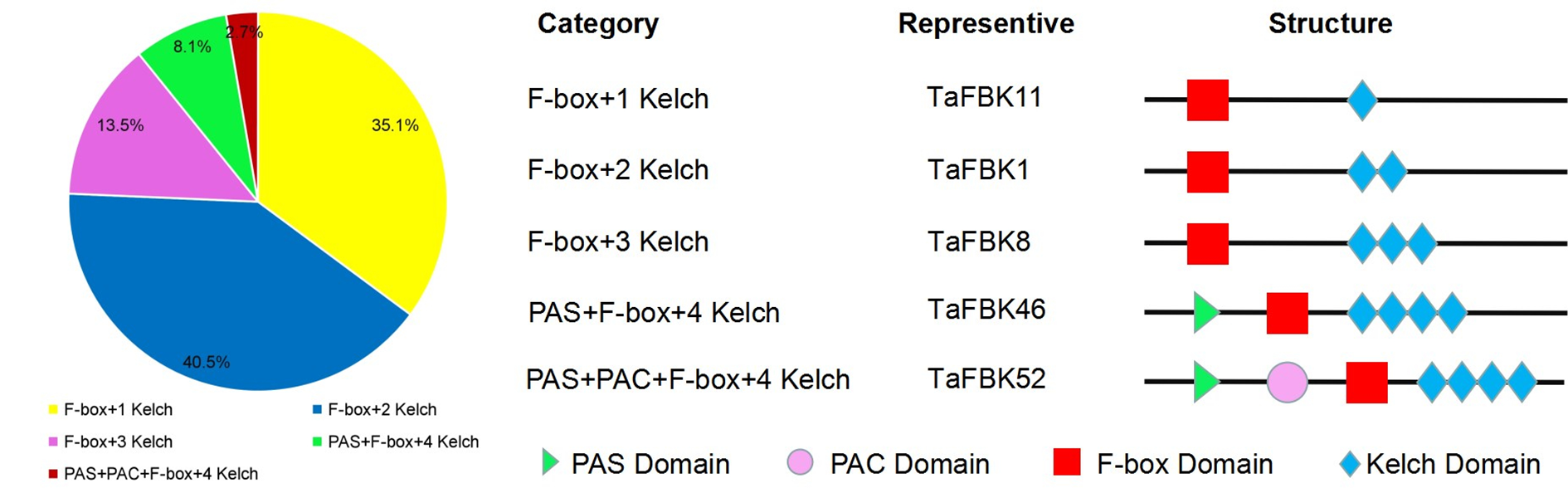

Supplement: S1 Fig — F-box, the protein with F-box domain; Kelch, F-box protein having Kelch domain; PAS, FBK protein with PAS domain that was named after three proteins that it occurs in: Per-period circadian protein, Arnt-Ah receptor nuclear translocator protein and Sim-single-minded protein; PAC, FBK protein with PAC domain that usually appears at the C-terminus of the PAS motif. (TIF) [file pone.0250479.s001.tif]

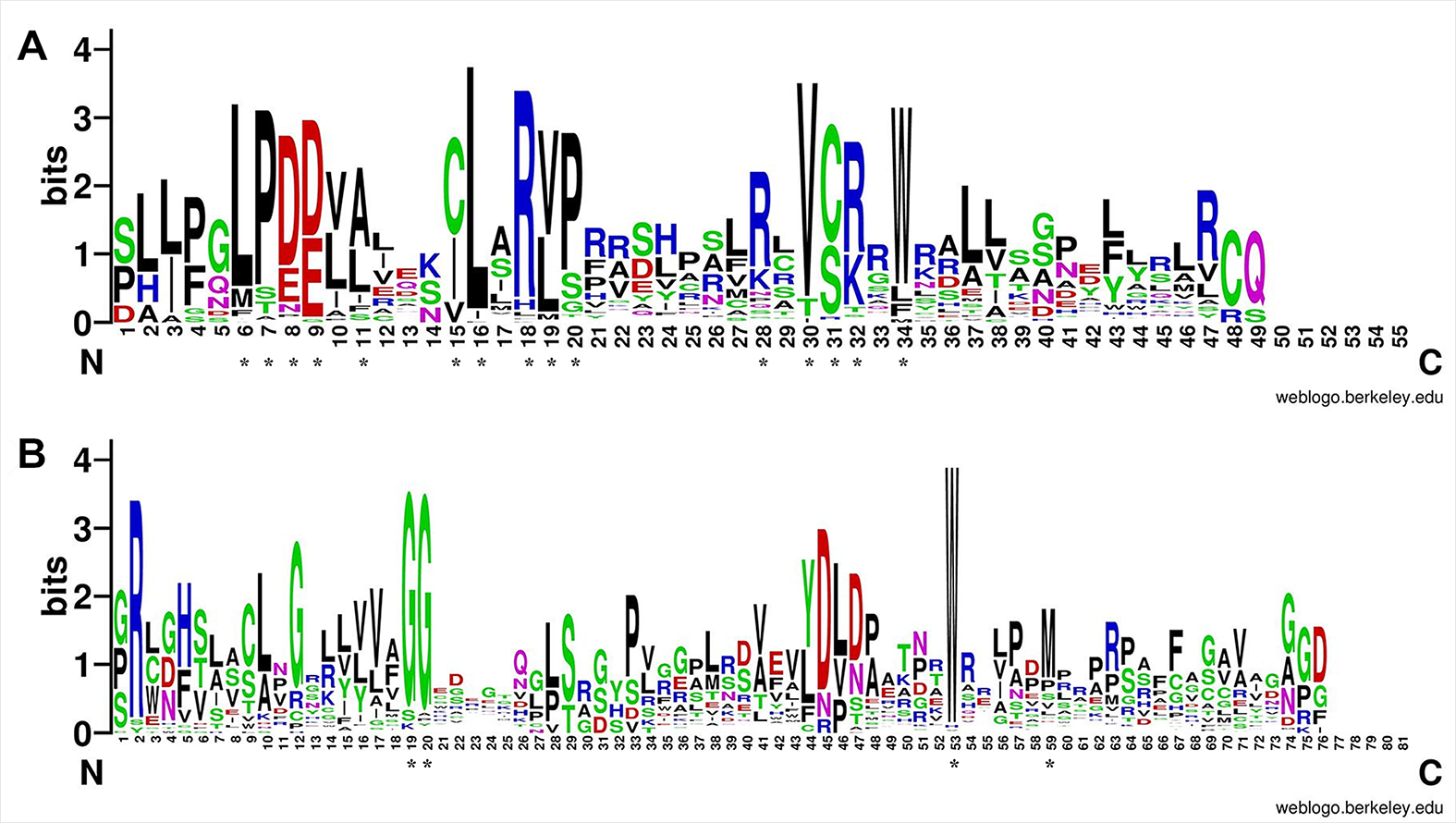

Supplement: S2 Fig — WebLogo generated by alignments of the F-box (A) or Kelch (B) domains of wheat FBKs. The F-box or Kelch motifs were retrieved from 74 wheat F-box proteins. The overall height of every stack is indicative of sequence conservation at the given position within the motif, whereas the height of the letters within each stack is indicative of the relative frequency of the corresponding amino acid. The bit score represents the information content for each position. Asterisks mark the conserved residues. (TIF) [file pone.0250479.s002.tif]

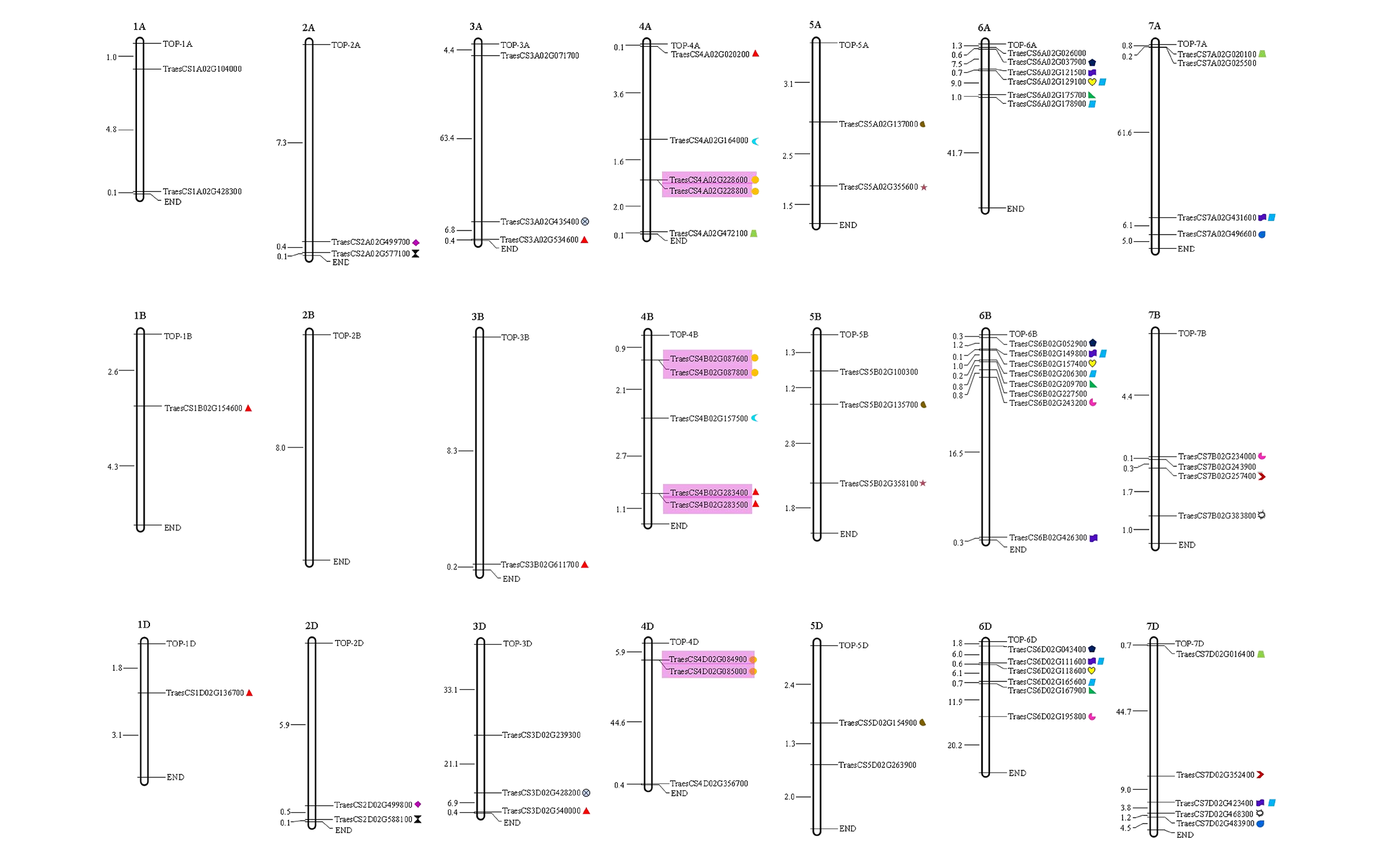

Supplement: S3 Fig — The chromosomes were drafted to proportion and the chromosome numbers were indicated at the top of each stave. Chromosomal distances were given in megabases (10 Mb). The gene names were listed at the right side of each chromosome corresponding to the position of each gene. Tandemly duplicated genes were shown in pink boxes. Segmental duplications were shown in colored blocks. (TIF) [file pone.0250479.s003.tif]
