# Supplementary material for: Genome-wide survey of the F-box/Kelch (FBK) members and molecular identification of a novel FBK gene TaAFR in wheat
Source: PLoS One. 2021 Jul 22;16(7):e0250479. doi: 10.1371/journal.pone.0250479 (PMC8298115; doi:10.1371/journal.pone.0250479)
Supplement: S1 Table — (DOC) [file pone.0250479.s004.doc]

**S1 Table. Primer data.**

| **Primer name** | **Sequence ( 5'→3' )** | **Product size** | **Usage of primer** |
| --- | --- | --- | --- |
| *TaAFR*-F | cccagaccagaaccagagcaag | 1327 bp | Gene amplification |
| *TaAFR*-R | TTACCAGGCATTGCTCTGAACT |
| RT-qPCR-*GAPDH*-F | CTGCCTTGCTCCTCTTGCTAA | 98 bp | RT-qPCR |
| RT-qPCR-*GAPDH*-R | CTTGATGGAAGGACCAGCAAC |
| RT-qPCR-*TaAFR-*F | CCTGTTCCGCACCGTCTCCT | 127 bp |
| RT-qPCR-*TaAFR-*R | GGCGAAGAGGAACGGCAGTGAGA |
| GFP-*TaAFR*-F | atacaccaaatcgactctagaATGAGCTTCTCGTCGGCGT | 1198 bp | GFP vector construction |
| GFP-*TaAFR*-R | ccggatccactagtatttaaatgtcgacGGCGTAGAGGACCTGCGC |
| *TaAFR*-*EcoR* I-F | GAATTCatgagcttctcgtcggcgtgcaagc | 1164 bp | Bait vector construction |
| *TaAFR*-*Sal* I-R | GTCGACtcaggcgtagaggacctgcgcgt |
| *TaAFR-F-box*-*EcoR* I-F | gaattcatgagcttctcgtcg | 225 bo |
| *TaAFR*-*F-box*-*Sal* I-R | GTCGACGGCGGCGGCCCCCGGC |
| *TaAFR*-*Kelch*-*EcoR* I-F | gaattcAAGGGCTCGGCTCCGC | 951 bp |
| *TaAFR*-*Kelch*-*Sal* I-R | GTCGACtcaggcgtagaggacctg |
| *TaRubisco*-*EcoR* I-F | CCATGGATGTCACCACAAAC | 1401 bp | Prey vector construction |
| *TaRubisco*-*EcoR* I-R | GGATCCTTTCCATACTTCACAAG |
| *TaSkp1*-*EcoR* I-F | GAATTCatggcggccgcgggagac | 540 bp |
| *TaSkp1*-*BamH* I-R | GGATCCctactcaaaggcccactg |
| *TaARL2-Nde I*-F | CATATGATGGGGCTCACGTT | 555 bp |
| *TaARL2*-*EcoR* I-R | GAATTCTTAGGACTTGCTGGC |
| *TaGV*-*EcoR* I-F | CGGAATTCATGACGGTGGT | 1217 bp |
| *TaGV*-*BamH* I-R | GGATCCCTAAAAGAAGAAAATAGATAGG |
| *TaRP*-*EcoR* I-F | GAATTCATGCCACTCGAACC | 342 bp |
| *TaRP*-*BamH* I-R | GGATCCTTACGCACTCTTTAAAG |
| *TaSLY1*-*Eco*R I-F | CGGAATTCATGGTGGCC | 1250 bp |
| *TaSLY1*-*Bam*H I-R | GGATCCTTACTGTGGTGGT |
| *TaNADH*-*Eco*R I-F | gaattcATGTCGACGACGGCGGGG | 357 bp |
| *TaNADH*-*Bam*H I-R | ggatccTCATTGGTCTTCTTTGCCATAGTT |
| *TaPOD*-*Eco*R I-F | GGAATTCATGACTCCGGCC | 984 bp |
| *TaPOD*-*Bam*H I-R | GGATCCTCAGTTGAACGCTG |
| *TaLRR*-*Nde* I-F | CATATGATGGCGCCTCG | 672 bp |
| *TaLRR*-*Eco*R I-R | GAATTCCTAGCATCCAAAGTCAT |
| *TaLac7*-*Nde* I-F | CGCATATGATGGCGCAGCTTTTGTG | 1661 bp |
| *TaLac7*-*EcoR* I-R | GAATTCCTAGCACACGGGCAGATCC |
| *TaCYP51*-*Eco*R I-F | CGGAATTCATGAGCCAGCAGG | 1181 bp |
| *TaCYP51*-*Bam*H I-R | GGATCCCTAGTTGTCGACGATGAG |
| *TaPAL*-*Eco*R I-F | CGGAATTCATGGAGTGCGAG | 2156 bp |
| *TaPAL*-*Bam*H I-R | GGATCCTCAGCAGAGCGGC |
| pSPY CE-*TaAFR*-F | tggcgcgccactagtggatccatgagcttctcgtcggcgt | 1188 bp | BiFC vector construction |
| pSPY CE-*TaAFR*-R | agcggtaccctcgaggtcgacGGCGTAGAGGACCTGCGC |
| pSPY NE-*TaSkp1*-F | cccaggcctactagtggatccatggcggccgcgggagac | 567 bp |
| pSPY NE-*TaSkp1*-R | agcggtaccctcgaggtcgacCTACTCAAAGGCCCACTGGTTC |
| pSPY NE-*TaSLY1*-F | cccaggcctactagtggatccatggtggccgccgcgctg | 1272 bp |
| pSPY NE-*TaSLY1*-R | agcggtaccctcgaggtcgacTTACTGTGGTGGTAGGTTGCTGC |
| pSPY NE-*TaARL2*-F | cccaggcctactagtggatccatggggctcacgttcacca | 585 bp |
| pSPY NE-*TaARL2*-R | agcggtaccctcgaggtcgacGGACTTGCTGGCAATGTTGC |
| pSPY NE-*TaPAL*-F | cccaggcctactagtggatccatggagtgcgagaccggc | 2181 bp |
| pSPY NE-*TaPAL*-R | agcggtaccctcgaggtcgacGCAGAGCGGCAGCGGCGC |
| pSPY NE-*TaCYP51*-F | cccaggcctactagtggatccatgagccagcaggaggtctacc | 1206 bp |
| pSPY NE-*TaCYP51*-R | agcggtaccctcgaggtcgacGTTGTCGACGATGAGCTTCCG |
| pSPY NE-*TaNADH*-F | cccaggcctactagtggatccatggaggccagtgaattcatgt | 384 bp |
| pSPY NE-*TaNADH*-R | agcggtaccctcgaggtcgacTTGGTCTTCTTTGCCATAGTTGTAG |
