# Supplementary material for: Genome-wide survey of the F-box/Kelch (FBK) members and molecular identification of a novel FBK gene TaAFR in wheat
Source: PLoS One. 2021 Jul 22;16(7):e0250479. doi: 10.1371/journal.pone.0250479 (PMC8298115; doi:10.1371/journal.pone.0250479)
Supplement: S4 Table — (DOC) [file pone.0250479.s007.doc]

**S4 Table. Screening of the candidate proteins interacting with TaAFR.**

| **No.** | **GenBank accession No.** | **Protein name** | **Protein abbreviation** | **Number of clones** | **Classification** | **References** |
| --- | --- | --- | --- | --- | --- | --- |
| 1 | AAX44976.1 | Ribulose bisphosphate carboxylase oxygenase | Rubisco | 15 | photosynthesis related protein | [1, 2] |
| 2 | XP_002456911.1 | photosystem Ⅱreaction center PSB 28 protein | PSB28 | 11 |
| 3 | AAP79890.1 | Skp1/ASK1-like protein | Skp1 | 1 | basal metabolism related protein | [3, 4] |
| 4 | XP_020187773.1 | NADH dehydrogenase [ubiquinone] 1 beta subcomplex subunit 9 | NADH | 3 |
| 5 | XP_015611313.1 | ADP-ribosylation factor 2-like isoform X1 | ARL2 | 1 | transport related protein | [5, 6, 7] |
| 6 | AK250591.1 | retrotransposon protein | RP | 3 |
| 7 | XP 0201683091 | SEC1 family transport protein SLY1 | SLY1 | 1 |
| 8 | XP_020183620.1 | Peroxidase 51-like | POD | 1 | stress resistance related protein | [8-12] |
| 9 | XP_014754759.1 | leucine-rich repeat protein 1 (LRR-8 Superfamily) | LRR | 1 |
| 10 | EMT13905.1 | Laccase-7 | Lac7 | 2 |
| 11 | Y09291.1 | obtusifoliol 14-alpha-demethylase | CYP51 | 1 |
| 12 | EMS66018.1 | Glucan endo-1,3-beta-glucosidase 14 | GV | 3 |
| 13 | CAE45949.1 | Unknown protein | — | 4 | — | — |

**References**

1. Yang LY, Han R. Effects of Ca^2+^ on wheat germination and seeding development under saline stress. Chinese Bulletin of Botany. 2011; 46: 155-161. doi: 10.3724/SP.J.1259.2011.00155
2. Tantray AY, Bashir SS, Ahmad A. Low nitrogen stress regulates chlorophyll fluorescence in coordination with photosynthesis and Rubisco efficiency of rice. Physiol Mol Biol Pla. 2020; 26: 83-94. doi: 10.1007/s12298-019-00721-0
3. Munné BS, Shikanai T, Asada K. Enhanced ferredoxin-dependent cyclic electron flow around photosystem I and a-tocopherolquinone accumulation in water-stressed ndhB-inactivated tobacco mutants. Planta. 2005; 222: 502-511. doi: 10.1007/s00425-005-1548-y
4. HajSalah El Beji I, Mouzeyar S, Bouzidi MF, Roche J. Expansion and functional diversification of SKP1-like genes in wheat (*Triticum aestivum* L.). Int J Mol Sci. 2019; 20: 3295–3312. doi: 10.3390/ijms20133295
5. McElver J, Patton D, Rumbaugh M, Liu CM, Yang LJ, Meinke DW. The TITAN5 gene of Arabidopsis encodes a protein related to the ADP ribosylation factor family of GTP binding proteins. Plant Cell. 2000; 12: 1379-1392. doi: 10.1105/tpc.12.8.1379
6. Toonen Ruud FG, Verhage M. Vesicle trafficking：pleasure and pain from SM genes. Trends Cell Biol. 2003; 13: 177-186. doi: 10.1016/S0962-8924(03)00031-X
7. Floor SN, Doudna JA. Get in LINE: Competition for newly minted retrotransposon proteins at the ribosome. Mol Cell. 2015; 60: 712-714. doi: 10.1016/j.molcel.2015.11.014
8. Beffa RS,Neuhaus JM,Meins F. Physiological compensation in antisense transformants: specific induction of an "ersatz" glucan endo-1,3-beta-glucosidase in plants infected with necrotizing viruses. Proc Natl Acad Sci USA. 1993; 90: 8792-879616. doi: 10.1073/pnas.90.19.8792
9. Hong SH, Lee SS, Chung JM, Jung HS, Singh S, Mondal S, et al. Site-specific mutagenesis of yeast 2-Cys peroxiredoxin improves heat or oxidative stress tolerance by enhancing its chaperone or peroxidase function. Protoplasma. 2017; 254: 327-334. doi: 10.1007/s00709-016-0948-0
10. Wang Q, Li G, Zheng K, Zhu X, Ma J, Wang D, et al. The soybean Laccase gene family: Evolution and possible roles in plant defense and stem strength selection. Genes. 2019; 10: 9. doi: 10.3390/genes10090701
11. Bianchet C, Wong A, Quaglia M, Alqurashi M, Gehring C, Ntoukakis V, et al. An Arabidopsis thaliana leucine-rich repeat protein harbors an adenylyl cyclase catalytic center and affects responses to pathogens. J Plant Physiol. 2019; 232: 12-22. doi: 10.1016/j.jplph.2018.10.025
12. Binjubair FA, Parker JE, Warrilow AG, Puri K, Braidley PJ, Tatar E, et al. Small-molecule inhibitors targeting sterol 14α-demethylase (CYP51): Synthesis, molecular modelling and evaluation against *Candida albicans*. ChemMedChem. 2020; 15: 1294-1309. doi: 10.1002/cmdc.202000250
