# Supplementary material for: Genome-wide survey of the F-box/Kelch (FBK) members and molecular identification of a novel FBK gene TaAFR in wheat
Source: PLoS One. 2021 Jul 22;16(7):e0250479. doi: 10.1371/journal.pone.0250479 (PMC8298115; doi:10.1371/journal.pone.0250479)
Supplement: S5 Table — (DOC) [file pone.0250479.s008.doc]

**S5 Table. Bioinformatics analyses of the candidate proteins interacting with TaAFR.**

| **Protein name** | **ORF length** | **Amino acid number** | **Molecular weight** | **Isoelectric point (*p*I)** | **Protein domain** | **Predicted subcellular localization** | **Signal peptide** | **Transmemb- rane region** |
| --- | --- | --- | --- | --- | --- | --- | --- | --- |
| Rubisco | 1389 bp | 463 aa | 51.18 kDa | 6.73 | RuBisCO-large | Chloroplast | no | no |
| Skp1 | 528 bp | 176 aa | 19.05 kDa | 4.33 | Skp1 | Nuclear | no | no |
| ARL2 | 546 bp | 182 aa | 20.67 kDa | 6.76 | ARF | Nuclear, Golgi | no | no |
| GV | 1200 bp | 400 aa | 44.25 kDa | 8.11 | Glyco_hydro_17 | Extracellular | yes | 381-398 aa |
| RP | 330 bp | 110 aa | 11.93 kDa | 10.02 | no | Extracellular | no | no |
| SLY1 | 1233 bp | 411 aa | 45.34 kDa | 5.60 | Sec1 | Cytoplasm | no | no |
| NADH | 345 bp | 115 aa | 13.36 kDa | 7.41 | Complex1_LYR | Mitochondrion | no | no |
| POD | 972 bp | 324 aa | 33.72 kDa | 7.86 | EF1_GNE | Plasma membrane | no | no |
| LRR | 660 bp | 220 aa | 23.82 kDa | 8.72 | LRRNT_2, LRR_1 | Plasma membrane | yes | no |
| Lac7 | 1647 bp | 549 aa | 57.99 kDa | 4.68 | Cu-oxidase | Extracellular | yes | no |
| CYP51 | 1167 bp | 389 aa | 44.57 kDa | 6.35 | p450 | Plasma membrane | no | no |
| PAL | 2145 bp | 715 aa | 77.04 kDa | 6.18 | Lyase_aromatic | Cytoplasm | no | no |
| PSB28 | 543 bp | 182 aa | 19.91 kDa | 10.01 | Psb28 | Chloroplast | yes | no |
