# Supplementary material for: Genome-wide survey of the F-box/Kelch (FBK) members and molecular identification of a novel FBK gene TaAFR in wheat
Source: PLoS One. 2021 Jul 22;16(7):e0250479. doi: 10.1371/journal.pone.0250479 (PMC8298115; doi:10.1371/journal.pone.0250479)

1. **IB (immunoblotting)**: IB was used to detect the expression of TaAFR, TaSkp1, TaARL2, TaPAL and GFP in the whole cell lysates (WCL) with HA or FLAG antibody.

1.1. Anti-FLAG

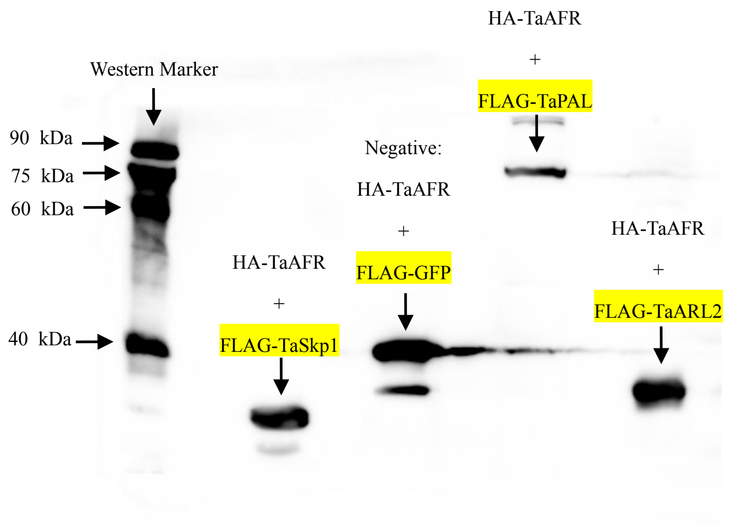

1.2. Anti-HA

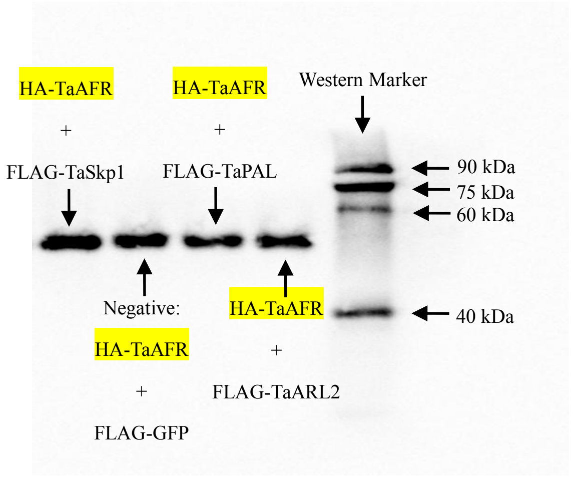

2. **IP (immunoprecipitated)**: The whole cell lysates were immunoprecipitated by HA-magnetic beads, then the eluted proteins were subjected to analyze with anti-FLAG antibody.

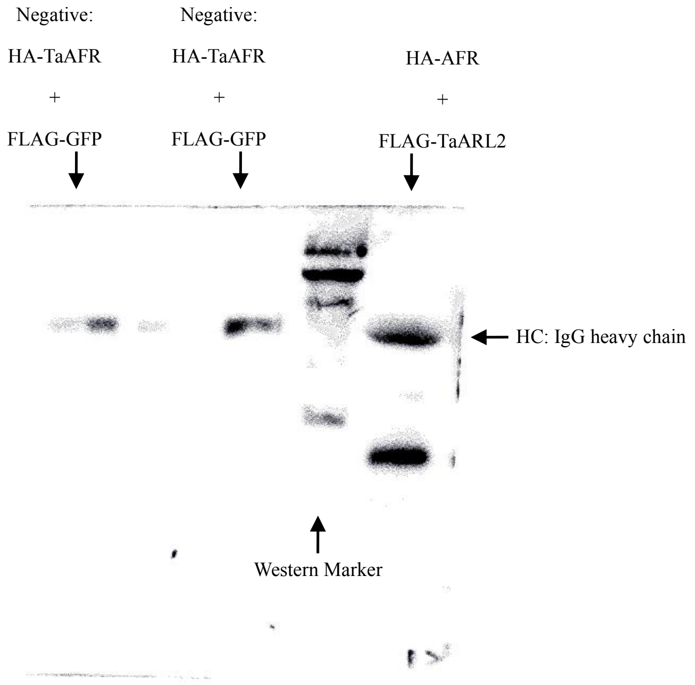

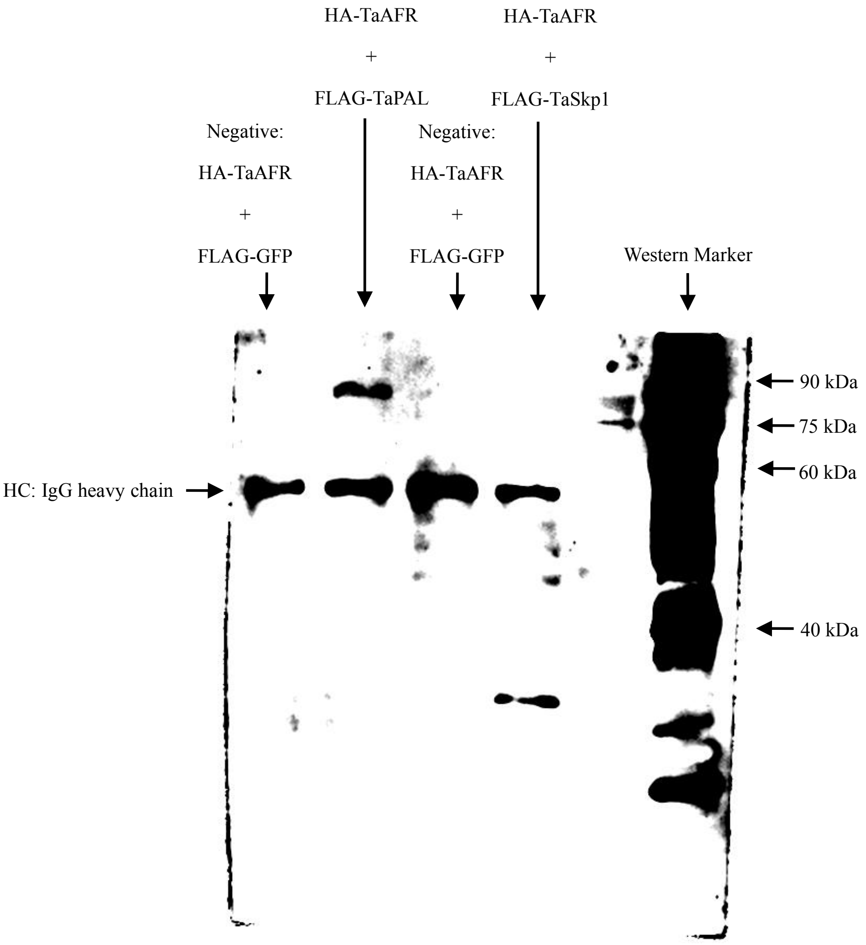

Supplement: S1 Raw images — (PDF) [file pone.0250479.s010.pdf]
